# Divergent and convergent evolution of housekeeping genes in human–pig lineage

**DOI:** 10.7717/peerj.4840

**Published:** 2018-05-24

**Authors:** Kai Wei, Tingting Zhang, Lei Ma

**Affiliations:** College of Life Science, Shihezi University, Shihezi, Xinjiang, China

**Keywords:** Housekeeping genes, Basal cellular function, Convergent evolution, Gene structure, Human–pig lineage

## Abstract

Housekeeping genes are ubiquitously expressed and maintain basic cellular functions across tissue/cell type conditions. The present study aimed to develop a set of pig housekeeping genes and compare the structure, evolution and function of housekeeping genes in the human–pig lineage. By using RNA sequencing data, we identified 3,136 pig housekeeping genes. Compared with human housekeeping genes, we found that pig housekeeping genes were longer and subjected to slightly weaker purifying selection pressure and faster neutral evolution. Common housekeeping genes, shared by the two species, achieve stronger purifying selection than species-specific genes. However, pig- and human-specific housekeeping genes have similar functions. Some species-specific housekeeping genes have evolved independently to form similar protein active sites or structure, such as the classical catalytic serine–histidine–aspartate triad, implying that they have converged for maintaining the basic cellular function, which allows them to adapt to the environment. Human and pig housekeeping genes have varied structures and gene lists, but they have converged to maintain basic cellular functions essential for the existence of a cell, regardless of its specific role in the species. The results of our study shed light on the evolutionary dynamics of housekeeping genes.

## Background

Housekeeping genes are typically genes that are consistently expressed across tissues and developmental stages for maintaining basic cellular functions, including basic metabolism, cellular transport and cell cycle (Butte, Dzau & Glueck, 2001; Zhu et al., 2008a). They have unique genomic features. For example, housekeeping genes have shorter structures (including the intron, coding sequence(CDS) and exon) compared with other genes (Eisenberg & Levanon, 2003; Vinogradov, 2004), their nucleotide composition is slightly richer in GC than that of tissue-specific genes (Vinogradov, 2003), and they have a reduced upstream sequence conservation (Farré et al., 2007; Bellora, Farré & Albà, 2007). Housekeeping genes are often considered as the minimal gene set needed for normal cellular physiology (Butte, Dzau & Glueck, 2001) and are widely used as internal controls for gene expression experiments and computational biology studies (Thellin et al., 1999; Robinson & Oshlack, 2010; Rubie et al., 2005; Vandesompele et al., 2002).

In previous studies, many human housekeeping gene sets have been identified. However, some sets slightly overlap. For example, only 155 genes were shared by three lists of microarray-defined housekeeping genes, including 501, 425 and 567 genes (Warrington et al., 2000; Hsiao et al., 2001; Eisenberg & Levanon, 2003). The low overlap may be explained by several reasons. Firstly, their complex transcriptional organisation may cause diverse definitions of housekeeping genes (Gingeras, 2007). Secondly, the expression of some genes may vary depending on experimental conditions (Greer et al., 2010). Why these genes vary across conditions needs further investigations. Thirdly, traditional techniques have their own drawbacks. For instance, microarray technology has a limited dynamic range and sensitivity and also suffers from poor detectability and reproducibility for low-copy and transiently expressed genes (Marioni et al., 2008; Fu et al., 2009; Bradford et al., 2010; Draghici et al., 2006).

RNA sequencing (RNA-seq) data greatly improve the detectability of housekeeping genes. For example, the amount of human housekeeping genes revisited by the RNA-seq data (3,804) has increased previous estimates based on microarray data (567) by sixfold (Eisenberg & Levanon, 2013). With advances in technology, large-scale RNA-seq has provided new insights into the definition of housekeeping genes. Some studies have suggested that transcripts should be used as housekeeping units, and all transcripts of a gene need to satisfy the criteria (Gingeras, 2007; Gerstein et al., 2007).

There is no consistent definition of human housekeeping genes. However, studying the genes of animals may be able to provide new information for housekeeping genes. Therefore, a comparative analysis of housekeeping genes between humans and other animals is of great interest. Human housekeeping genes are commonly used as control genes in real-time quantitative polymerase chain reaction (qRT-PCR) for other animals. However, whether human genes can be used as references for other animals remains unclear. For instance, the most commonly used human reference genes (e.g., *ACTB* and *GAPDH*) do not always apply to all tissues of different organisms (Brattelid et al., 2010; Kozera & Rapacz, 2013). Therefore, to well define a housekeeping gene set in another animal may be valuable. More importantly, housekeeping genes show very strict conservation in the evolutionary process, so the comparison of evolutionary dynamics will allow a fundamental understanding of evolutionary biology.

As an important meat resource for humans, the pig (*Sus scrofa*) is a well-studied organism. Given the anatomical similarities with humans, pigs are often used as a biomedical model in research (Lunney, 2007; Rolandsson et al., 2002; Lee et al., 2009; Becker et al., 2010). Surveying pig housekeeping genes may help pave the way for a greater understanding of the basal mechanisms that maintain cell function. In the present study, we identified housekeeping genes in pig using RNA-seq data and then compared their structure and function with human housekeeping genes. In addition, we discussed the impact of selection pressure and convergent evolution on the functional conservation of housekeeping genes. The present study provided detailed information on pig housekeeping genes and their functional features and offered insights into their evolutionary dynamics.

## Materials and Methods

### Data preparation

To define housekeeping gene sets, gene expression datasets were downloaded from the Sequence Read Archive (SRA) database of the National Center for Biotechnology Information (NCBI, September 2016) (Kodama, Shumway & Leinonen, 2012). In addition, pig genomic annotation (*Sus Sscrofa* 10.2) was downloaded from the Ensembl Genome Browser (September 2016) (Kinsella et al., 2011). The RNA-seq dataset of 14 experiments were used to identify housekeeping genes, which were derived from 21 tissues (heart, spleen, liver, kidney, lung, musculus longissimus dorsi, occipital cortex, hypothalamus, frontal cortex, cerebellum, endometrium, mesenterium, greater omentum, backfat, gonad, ovary, placenta, testis, blood, uterine and lymph nodes), containing a total of 131 samples (Table S1). The SRA files were downloaded from NCBI and then converted to fastq files by using fastq-dump (Kodama, Shumway & Leinonen, 2012). RNA-seq reads were then filtered by IlluQC.pl (Patel & Jain, 2012) whilst requiring an average read quality above 20. Then, the reads were aligned to a pig genome sequence (Sus Sscrofa10.2) using TopHat (Trapnell, Pachter & Salzberg, 2009; Külahoglu & Bräutigam, 2014; Ghosh & Chan, 2016). The alignments were then fed to an assembler Cufflinks (Trapnell, Pachter & Salzberg, 2009) to assemble aligned RNA-seq reads into transcripts and estimate their abundances, which were measured in fragments per kilobase of exon per million fragments mapped.

### Definition of housekeeping genes

Housekeeping genes were defined according to the following criteria: (i) the transcripts could be detected in all 21 tissues (6,072 transcripts); (ii) the transcripts showed low expression variance across tissues: *P* > 0.1 (4,068 transcripts; Kolmogorov–Smirnov test); (iii) no exceptional expression in any single tissue; that is, the expression values were restricted within the fourfold range of the average across tissues (3,914 transcripts); and (iv) all transcripts of a housekeeping candidate gene met the above criteria (3,136 genes).

### Structure analysis

The structure data of genes were obtained from the Ensembl BioMart (Kinsella et al., 2011). Human housekeeping genes were derived from the reference (Eisenberg & Levanon, 2013), considering their similar type of data from RNA-seq and stringency of the definition by expression breadth and stability. A total of 3,136 and 3,804 housekeeping genes of pigs and humans were obtained, respectively. The length of various parts of housekeeping genes were compared by Mann–Whitney test (Table 1). In addition, the length of various parts of 3,000 non-housekeeping genes were also compared by random selection in humans and pigs.

**Table 1:** Comparison of housekeeping and non-housekeeping genes between pigs and humans.

Structure	Housekeeping gene			Non-housekeeping gene		
	Pigs	Humans	*P*-value^c^	Pigs	Humans	*P*-value
Total intron length^a^	28,108 ± 173^b^	21,062 ± 297	1.5e^−105^	5,9318 ± 523	47,216 ± 487	2.7e^−56^
5′ UTR length	156 ± 3	125 ± 1.5	3.7e^−34^	207 ± 4.5	234 ± 4.1	1.6e^−29^
3′ UTR length	658 ± 13	549 ± 5	1.4e^−73^	958 ± 7.3	558 ± 4.2	7.3e^−65^
Average exon length per gene	261 ± 3	227 ± 1	1.8e^−6^	249 ± 2.7	265 ± 2.9	2.4e^−33^
CDS length	2,181 ± 10	1,460 ± 5	8.7e^−234^	3,047 ± 11.4	3,124 ± 10.8	3.1e^−17^
Transcript length	3,312 ± 13	2,200 ± 5	7.7e^−7^	4,021 ± 17.1	3,841 ± 14.3	8.6e^−94^
Number of exons	9.2 ± 0.1	8.8 ± 0.2	1.7e^−4^	15.2 ± 0.2	13.6 ± 0.1	4.2e^−4^

**Notes.**

a The length is measured in nucleotides.

b The value gives the average and standard error of mean.

c The *P*-value was calculated based on the Mann-Whitney test.

UTR untranslated region CDS coding sequence

### Gene Ontology (GO) analysis

The analysis of functional annotations of housekeeping genes was performed using DAVID, ver. 6.7, available on their website (Huang da, Sherman & Lempicki, 2009a; Huang da, Sherman & Lempicki, 2009b). All expressed genes in the data were used as background. Comparative analysis of housekeeping and non-housekeeping genes between humans and pigs was performed. The false discovery rates (FDR) were calculated to estimate the extent to which genes were enriched in GO categories (Ashburner et al., 2000). Probabilities less than 0.01 were used as the cut-off value and considered to show a significant level of correlation. Heat map analysis was also conducted through DAVID to visualise a matrix of enriched GO.

### Evolutionary features analysis

Evolutionary features of housekeeping and non-housekeeping genes between humans and pigs were compared by calculating the substitution ratio. The number of non-synonymous substitutions per non-synonymous site (dN) and the number of synonymous substitutions per synonymous site (dS) were estimated using the Nei–Gojobori method embedded in MEGA 7.0 (*Z*-test, *P* < 0.05) (Kumar, Stecher & Tamura, 2016; Nei & Kumar, 2000). From the Scope row, select the Overall Average option. For the Gaps/Missing data treatment option, select Pairwise Deletion. The genome sequences of orthologous genes were downloaded from Ensembl BioMart. The dN/dS ratios were calculated to assess the selection pressure (Hurst, 2002; Yang & Nielsen, 2002; Dasmeh et al., 2014). Information of active sites of proteins was obtained from UniProt Knowledgebase (Boutet et al., 2016; Pundir et al., 2015). Species-specific housekeeping genes that have similar functions were processed to search for their active sites.

## Results

### Gene expression profile

**Figure 1:**
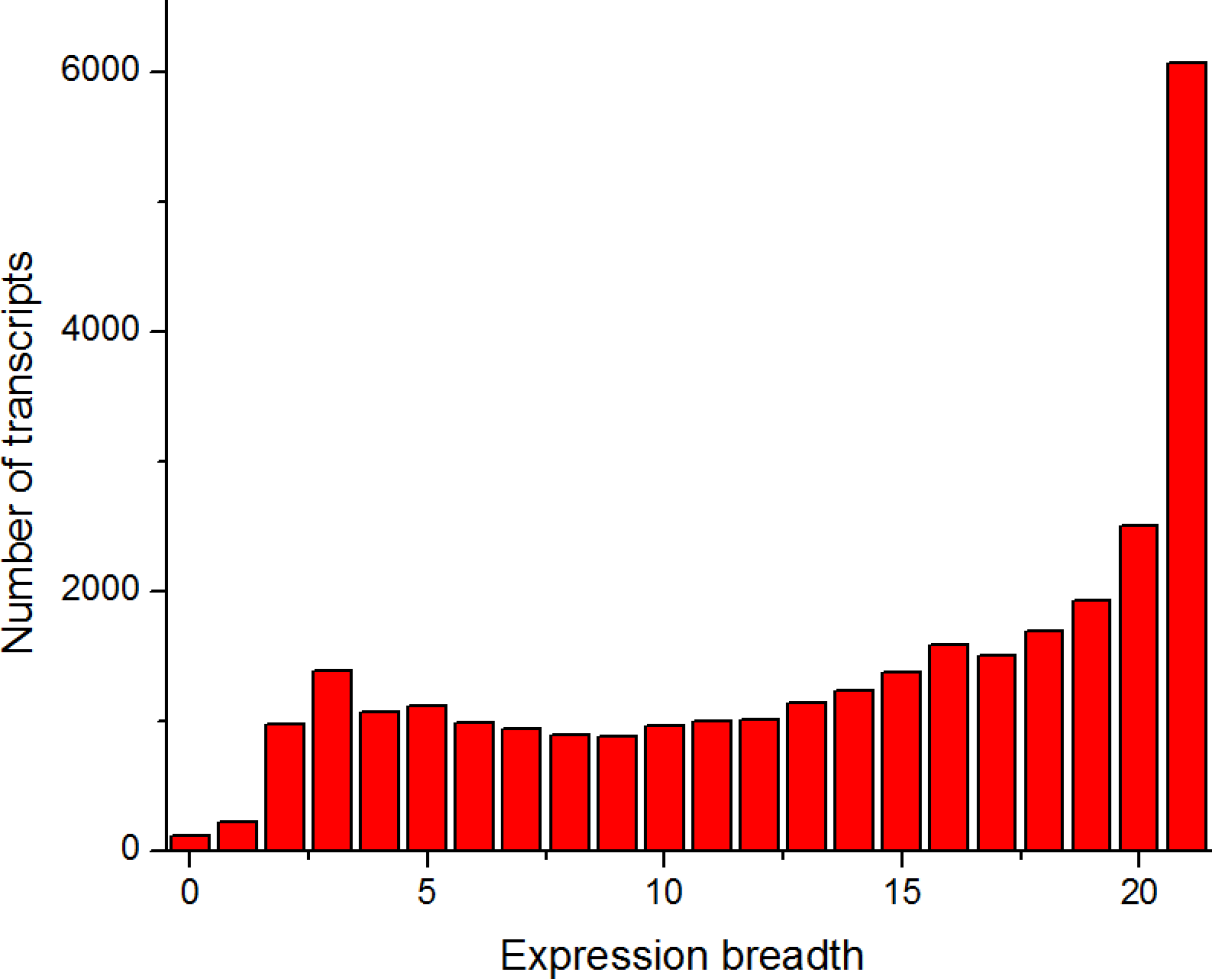
Number of tissues where a given transcript was detected. The expression breadth (horizontal axis) denotes the number of tissues where a given transcript was detected. The zero value of the expression breadth indicates undetected transcripts.

To identify the housekeeping genes in pigs, we surveyed the expression distribution of 30,585 transcripts across 21 tissues of pigs (see Methods, Fig. 1 and Fig. S1). The detectability of RNA-seq data was high, and only 116 transcripts were undetected in the present study. The 226 transcripts showed tissue-specific expression (expressed in one tissue), whereas 6,072 transcripts were found to be broadly expressed in all 21 tissues (Fig. 1). This finding was consistent with the expression tissue breadth of human genes (Zhu et al., 2008a; Zhu et al., 2008b; Eisenberg & Levanon, 2013).

### Identification of pig housekeeping genes

To obtain the transcripts with ubiquitous expression level across pig tissues, we selected 6,072 transcripts detected in 21 tissues as candidates. The background differences between different sequencing projects resulted in a batch effect between samples, including the difference in sequencing depth and coverage. Therefore, we chose a single sequencing project to assess the uniformity of gene expression. Furthermore, the expression uniformity of candidates in the ERP002055 sequencing project was evaluated using the Kolmogorov–Smirnov test and was accessed using the *P*-value (Farajzadeh et al., 2013). Figure S2 shows the frequencies of candidates with *P*-value greater than the given cutoff. Approximately 67% of all candidates had *P*-values greater than 0.1, implying that their expression levels did not significantly vary across tissues and had a high level of expression uniformity. Therefore, we defined the cutoff of the uniform level as *P >* 0.1 for the following analyses, which resulted in a list of 4,068 unique transcripts, belonging to 3,754 genes. The housekeeping gene was further restricted into the gene whose transcripts passed the criteria. Altogether, 3,136 genes passed the restriction (File S1), approximately a third of which were unannotated, and 356 genes in pigs possess no orthologues in humans. In addition, housekeeping genes showed a significantly lower number of transcripts (1.22 transcripts on average) compared with whole genes in pig (1.84 transcripts on average) (Mann–Whitney test, *P* < 0.05). Housekeeping genes are always stably expressed in any tissue and environmental condition, but non-housekeeping genes, especially tissue-specific genes, may adjust to different conditions by different transcript isoforms.

**Figure 2:**
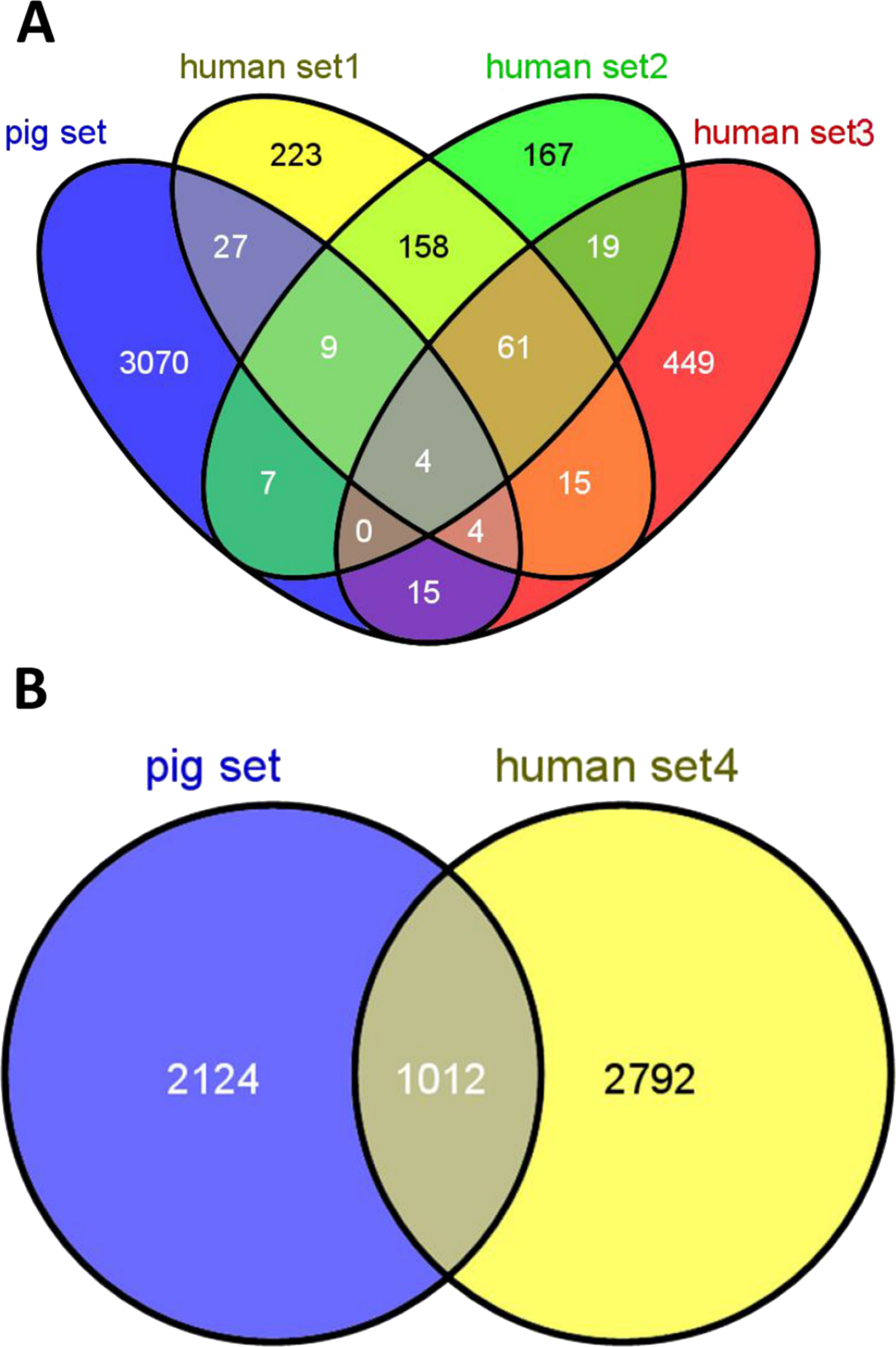
Overlap of housekeeping genes between pigs and humans. Overlap of pig housekeeping gene set identified in the present study (A) with three human gene sets identified by microarray data (Warrington et al., 2000; Hsiao et al., 2001; Eisenberg & Levanon, 2003) and (B) with a human set identified by RNA-seq data (Eisenberg & Levanon, 2013).

Figure 2 shows the overlap of pig housekeeping genes identified in the present study with previously reported human housekeeping genes (Warrington et al., 2000; Hsiao et al., 2001; Eisenberg & Levanon, 2003; Eisenberg & Levanon, 2013). In addition, a lower overlap rate of housekeeping genes between pigs and humans was observed and showed significant difference with any two random sets of genes from pigs and humans (*T* test, *P* < 0.01). To accurately describe the features, housekeeping genes were grouped into three sets of genes, namely, common housekeeping genes observed in pigs and humans, human-specific housekeeping genes and pig-specific housekeeping genes. We obtained 1,012 common, 2,792 human-specific and 2,124 pig-specific housekeeping genes (Fig. 2B).

### Structural comparison of housekeeping genes between pigs and humans

**Figure 3:**
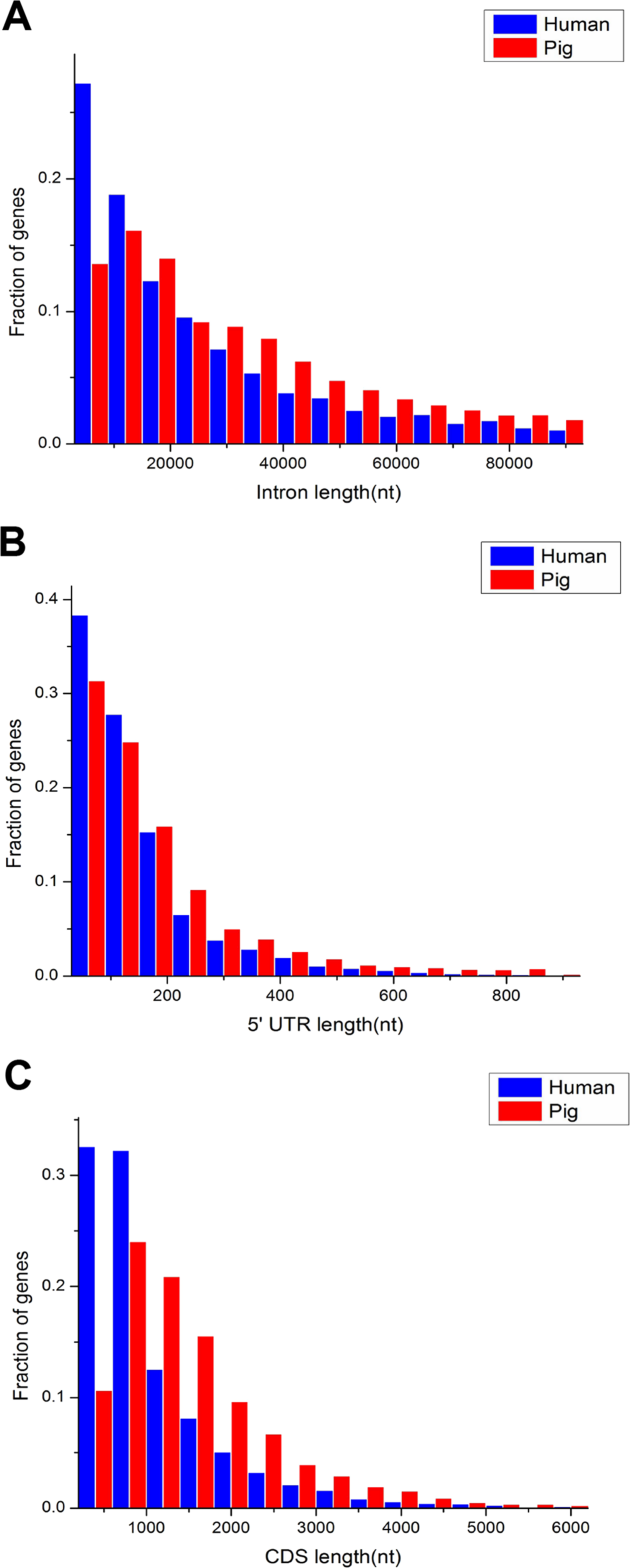
Comparison of length distribution of housekeeping gene structures between pig and human. nt, nucleotide(s); 5′ UTR, 5′ untranslated region (UTR); CDS, coding sequence.

The comparison of length distribution of total intron, 5′ untranslated region (UTR) and CDS in homologous housekeeping genes shows that pig genes have a long length, whereas human genes have a short length (Figs. 3A–3C). Furthermore, Table 1 shows the average lengths of various structures of the housekeeping and non-housekeeping genes that correspond to one another in pigs and humans. All structures of pig housekeeping genes were significantly longer than human housekeeping genes (Table 1), indicating that human housekeeping genes hold a greater impact of gene structure, which were consistent with the previous analyses of pig genomes (Groenen et al., 2012). This finding implied that different purifying selection pressures were applied between pigs and humans, showing that selective pressure may render genes as short as possible for reducing the cost in the transcription process (Ucker & Yamamoto, 1984; Castillo-Davis et al., 2002). Although the structural length of non-housekeeping genes showed a significant difference, non-housekeeping genes do not show consistent structural features unlike housekeeping genes. For example, the total intron length, 3′ UTR length and transcript length are longer in pigs than in humans, but the 5′ UTR length, average exon length and CDS length are shorter in pigs than in humans (Table 1).

### Evolutionary dynamics of housekeeping genes

Evolutionary features of housekeeping genes may provide a deeper understanding of the evolutionary trend of housekeeping genes in different species. For the maintenance of essential function, housekeeping genes are thought to evolve more slowly than other genes (Zhang & Li, 2004). To investigate this feature, the number of non-synonymous substitutions per non-synonymous site (dN), the number of synonymous substitutions per synonymous site (dS) and dN/dS ratio were calculated for pig and human housekeeping genes using mouse (*Mus musculus*) as an outgroup (Files S2 and S3). In addition, the phylogeny of the mouse is close to pigs and may even be closer to humans (Meredith et al., 2011). Thus, we also selected elephant (*Loxodonta africana*) as an outgroup to calculate for dN, dS and dN/dS for pig and human housekeeping genes (Files S4 and S5). Generally, synonymous substitutions occur randomly, which may not or slightly suffer from selection pressure and do not appear to change the gene function, but non-synonymous substitutions do not occur randomly, which may be caused by strong selection pressure and change the function of housekeeping genes (Nei & Kumar, 2000; Kimura, 1983).

In evolutionary analysis, the housekeeping genes between pigs and humans showed significant difference with mouse and elephant as outgroups (Table 2). However, statistical differences were only observed in the dS of non-housekeeping genes between pigs and humans with mouse and elephant as outgroups (Table S2). The selection pressure of non-housekeeping genes between pigs and humans did not show a significant difference. This result may indicate that housekeeping genes show a specific evolutionary feature related to non-housekeeping genes.

**Table 2:** Evolutionary features of housekeeping genes.

Terms	Mouse^a^			Elephant		
	Pigs	Humans	*P*-value^c^	Pigs	Humans	*P*-value
dN	0.084 ± 0.012^b^	0.065 ± 0.01	0.003	0.085 ± 0.010	0.058 ± 0.009	0.001
dS	0.82 ± 5.57	0.70 ± 5.12	0.001	0.76 ± 6.32	0.63 ± 4.67	0.001
dN/dS	0.12 ± 0.011	0.10 ± 0.014	0.004	0.14 ± 0.023	0.11 ± 0.020	0.003

**Notes.**

a Mouse and elephant are outgroups.

b The value gives the average and standard error of mean.

c The *P*-value was calculated based on the Mann-Whitney test.

**Figure 4:**
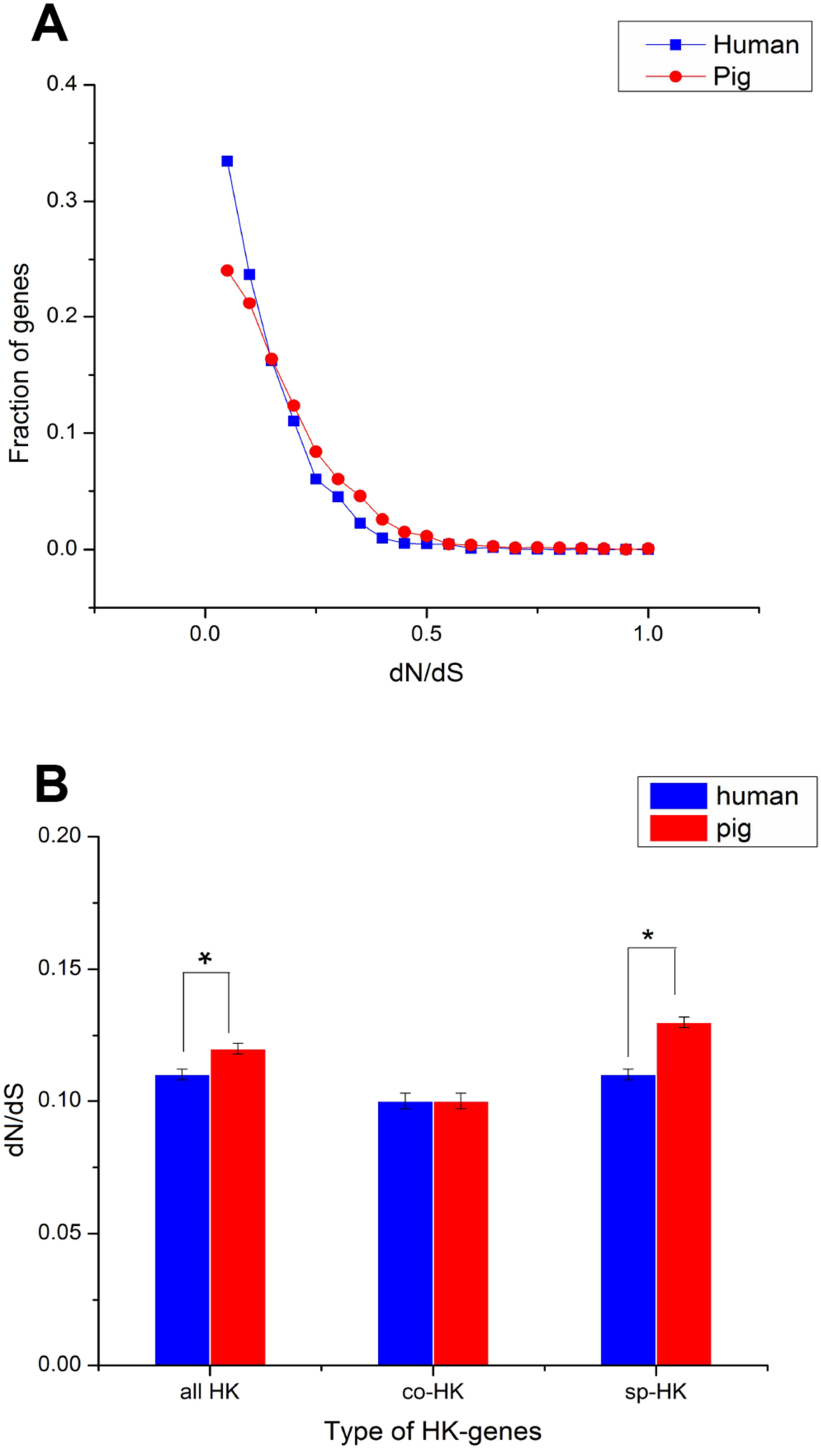
Purifying selection on housekeeping genes with mouse as outgroup. (A) The distribution of the dN/dS ratio. (B) The dN/dS ratios of total (all HK), common (co-HK) and species-specific (sp-HK) housekeeping genes were compared between pig and human (Mann–Whitney test, * denoted *P* < 0.05), respectively.

The dN followed a power law distribution similar to that of dN/dS with mouse and elephant as outgroups (Fig. 4A, Figs. S3A, S4A and S5A), displaying a relatively large number of genes with a few non-synonymous substitutions and a small fraction of genes with several substitutions (Fig. 4A and Fig. S4A). In addition, most dN/dS ratios were lower than 1, implying that purifying selection acted on the housekeeping genes to ensure the stability of most genes’ functions. The lesser the dN/dS ratio, the stronger the purifying selection. Furthermore, the purifying selection pressure on housekeeping genes was slightly stronger in humans than in pigs (Fig. 4 and Fig. S4).

Although mouse as outgroup showed similar results with elephant as outgroup, but with a lower difference when mouse and elephant is used as the group, respectively (Mann–Whitney test, *P* < 0.05). This result might be caused by the close phylogenetic relationship of mouse and humans (91 Myr ago) compared with pigs (97 Myr ago) and the long phylogenetic time of humans and pigs compared with elephant. Thus, a small difference was obtained when elephant was used as outgroup.

**Figure 5:**
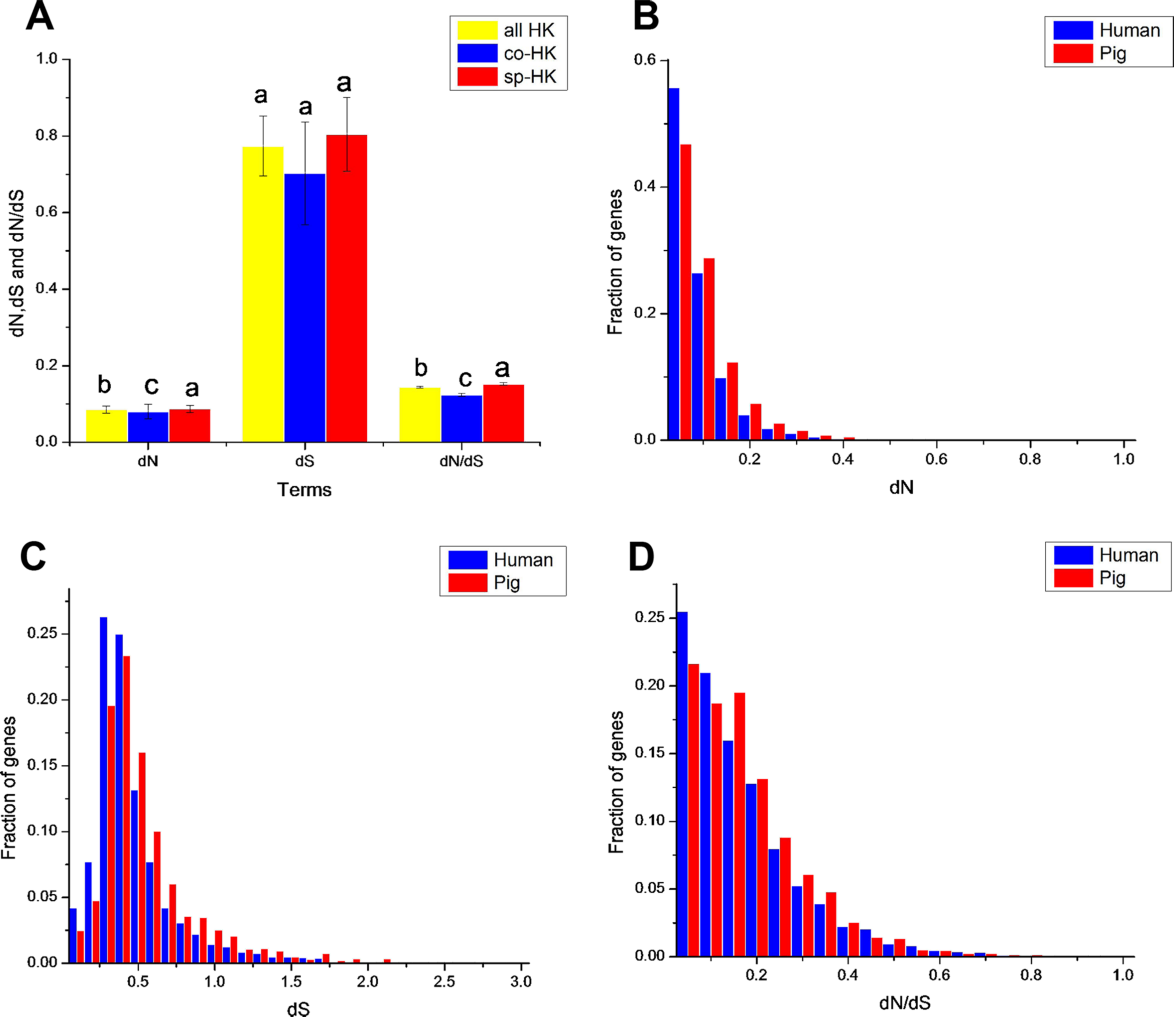
Comparison of evolutionary features of housekeeping genes with mouse as outgroup. (A) dN, dS and dN/dS of all, common and species-specific pig housekeeping genes were compared based on the Mann–Whitney test. In a signal cluster, all such means that share a common English letter are similar; otherwise, they differ significantly at *P* < 0.05. (B)–(D) Distributions of dN, dS and dN/dS of species-specific housekeeping genes in pigs and humans.

The dN/dS ratios of common housekeeping genes showed no difference between pigs and humans, but the ratios of species-specific housekeeping genes were significantly lower in humans than in pigs (Mann–Whitney test, *P* < 0.05) (Fig. 4B and Fig. S4B). Furthermore, for both humans and pigs, the dN/dS ratios of common genes were significantly lower than those of species-specific genes (Fig. 5A, Figs. S6 and S7). This result suggested that common housekeeping genes suffered a more stringent purifying selection to remove alleles than species-specific genes.

Moreover, the results of the dN/dS ratios (or dN) also implied that human housekeeping genes have evolved more stable than pig housekeeping genes because the substitution ratio was significantly lower in humans than in pigs (Table 2 and Figs. 5B–5D). This result may indicate that pig housekeeping genes may have wider evolutionary potential than human housekeeping genes. The dS of human species-specific genes had lower values than that of pig genes (Fig. 5C), showing that human housekeeping genes undergo a slower neutral evolution than pig housekeeping genes.

The dS followed an approximately normal distribution (Figs. S3B and S5B), which occurred around a central value (0.77 and 0.63 in pig and human housekeeping genes with mouse as outgroup, respectively). This finding implies the random tendency of synonymous substitutions. No significant difference was noted in the synonymous substitutions between common and species-specific genes within a species (Fig. 5A, Figs. S6 and S7).

### Associated function of housekeeping genes

**Figure 6:**
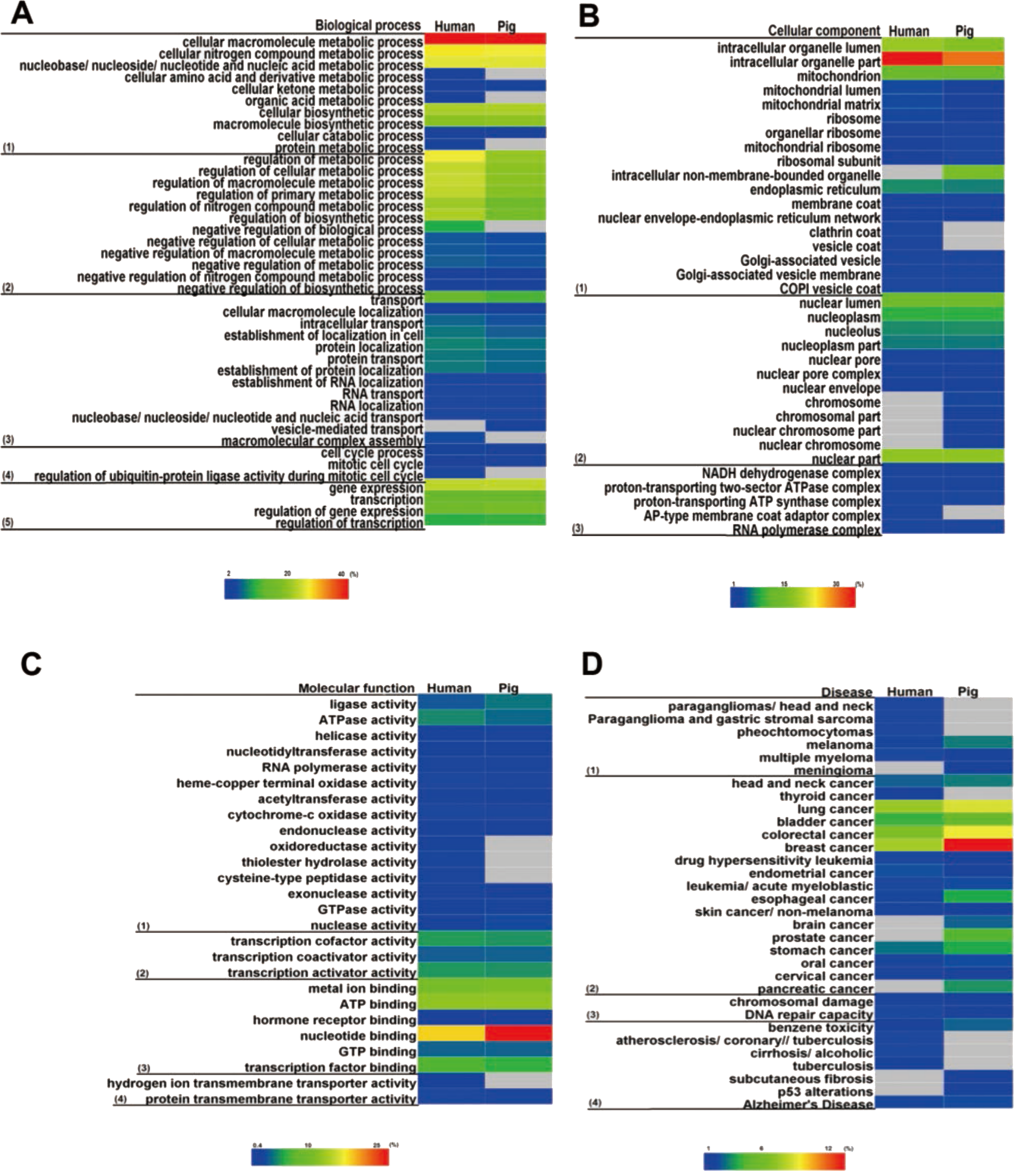
Functional enrichment analysis for housekeeping genes. Housekeeping genes were enriched in GO categories of (A) biological process, (B) cellular component, (C) molecular function, and (D) disease. Colour bars show gene frequency from 0. The basal cellular function between pigs and humans showed high consistency. (A): (1) Biological process categories included the basal metabolism, (2) regulation of metabolic processes, (3) cellular transport, (4) cell cycle, and (5) gene expression and regulation. (B): (1) Cellular component categories included organelle, (2) nuclear, and (3) micromolecular complex. (C): (1) Molecular function categories included catalytic activity, (2) transcription factor activity, (3) binding activity, and (4) transporter activity. (D): (1) Disease categories included tumour, (2) cancer, (3) chromosomal damage and repair, and (4) other disease.

We then characterised the housekeeping genes that enriched the molecular function, biological process, cellular component and disease based on DAVID. The heat map shown in Fig. 6 illustrates the similar enrichment of housekeeping genes between pigs and humans. Briefly, housekeeping genes were predominantly detected as genes associated with GO terms related to basal metabolism that are indispensable for cellular physiology, indicating that housekeeping genes are essential for basic physiological processes (Fig. 6). However, the non-housekeeping genes are mainly associated with the differentiation, development and specific functions of specific tissues or organs (Table S3). This finding shows that humans and pigs have similar basic cellular functions. Although some differences in disease enrichment were noted, many common diseases were found between humans and pigs.

Of note, many pig housekeeping genes were enriched in human diseases, especially in several cancers with high mortality rates: breast cancer, lung cancer and colorectal cancer (Fig. 6D). This finding may be beneficial for studies of human diseases (Tu et al., 2006), given that pigs do not possess some human high risk genes. For instance, alcohol-induced cirrhosis was enriched in human housekeeping genes, but not in pigs.

### Functional convergence

Interestingly, the functional enrichment analyses showed a coherent trend in pig and human housekeeping genes, although low overlap of gene lists and differences in gene structure between the two species were found. For example, for biological process, pigs and humans showed a slight difference in GO term enrichment (Fig. 6A). In addition, similar trends were observed in the active molecules related to basic metabolism and gene expression (Figs. 6B and 6C).

**Figure 7:**
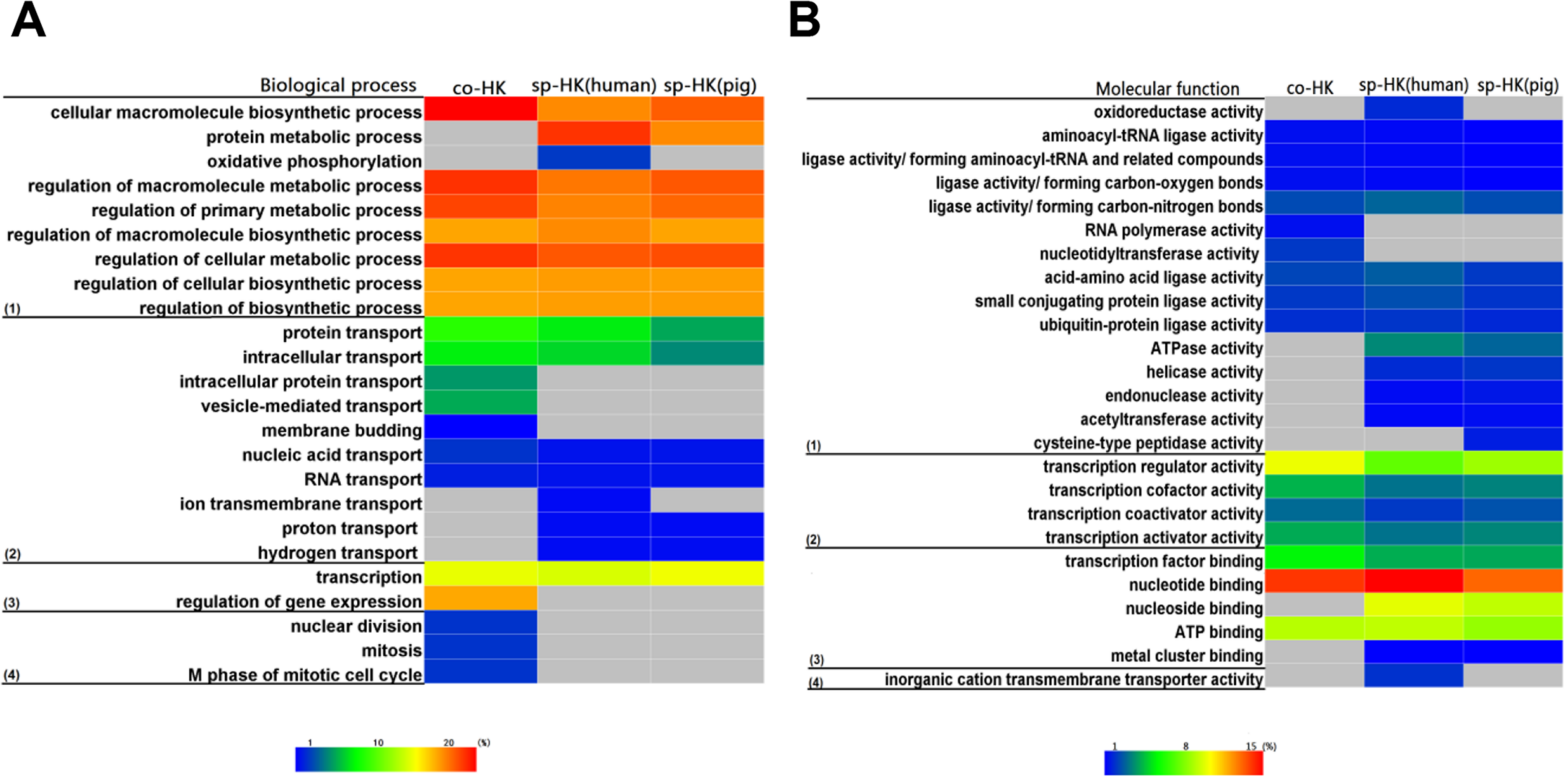
Comparison of functional enrichment analysis. When we compared functional enrichment, common housekeeping genes (co-HK) showed significant difference with species-specific housekeeping genes (sp-HK), but the sp-HK genes between pigs and humans showed very high consistency. Colour bars show gene frequency from 0. (A): (1) Biological process categories included the basal metabolism and regulation, (2) cellular transport, (3) gene expression and regulation, and (4) nuclear division. (B): (1) Molecular function categories included catalytic activity, (2) transcription factor activity, (3) binding activity, and (4) transporter activity.

The above analysis revealed that the functions of pig and human housekeeping genes were consistent, implying that the selection pressure may preclude the species differentiation of housekeeping genes for the maintenance of basal cellular functions, especially for species-specific housekeeping genes. To confirm this conjecture, we performed functional enrichment analysis for common and species-specific housekeeping genes. The heat map shown in Fig. 7 illustrates the higher similarity between two species-specific terms than between common and species-specific terms. These results indicated housekeeping genes suffered strong selection pressure for maintaining normal life activities, and human and pig species-specific housekeeping genes converged on the basal cellular function.

### Mechanistic convergence

**Figure 8:**
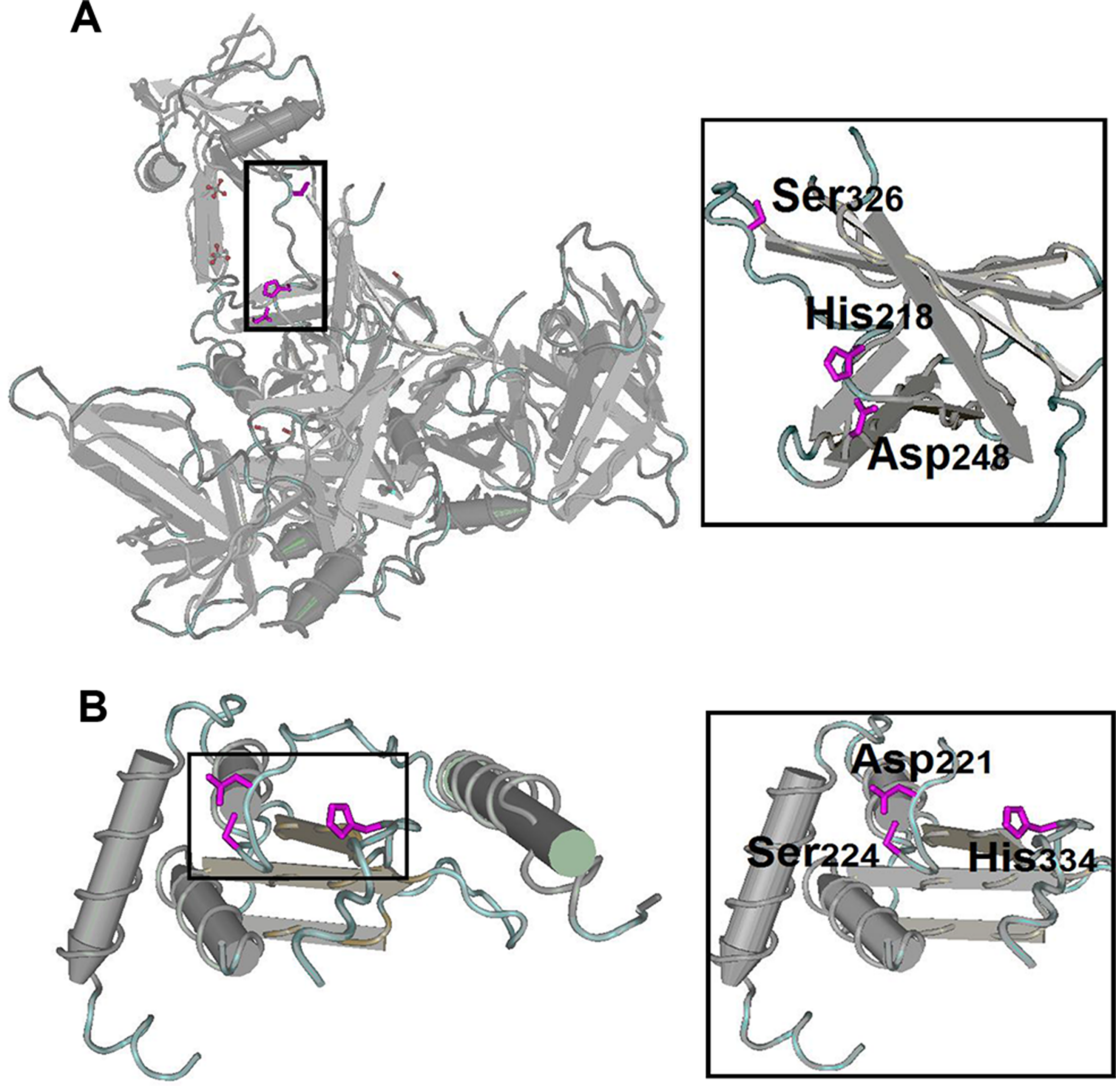
Structures of the ‘classical’ Ser/His/Asp triad configuration. (A) Serine protease HTRA4 from pigs. (B) OTU domain-containing protein 5 from humans. A zoomed-in view of the catalytic domain is shown to the right of each structure. The side chains of Ser/His/Asp triad are shown in principle.

To understand the mechanistic constraints on the function of housekeeping proteins, we analysed the evolutionary constraints on protein structure, active site feature and chemical reaction centre. We found some similar active site features in housekeeping peptidases (Fig. 8, Table 3), which reflected the intrinsic chemical constraints on enzymes, leading evolution to independently converge on equivalent solutions repeatedly (Buller & Townsend, 2013; Dodson & Wlodawer, 1998). As housekeeping genes mainly perform basic metabolic pathways of cells and peptidases are the main enzymes that perform these functions, we chose peptidases to study mechanistic convergence. The chemical and physical constraints on enzyme catalysis have caused identical triad arrangements in housekeeping peptidases in the human–pig lineage, such as classical catalytic Ser/His/Asp triad and non-classical variants (Table 3). However, the peptide sequences and their 3D structural profiles totally differed from each other (Figs. 8A and 8B). The classical Ser/His/Asp catalytic triad is a universal phenomenon in the serine protease class (E.C. 3.4.21), where serine is the nucleophile, histidine is the general base or acid, and aspartate helps orient the histidine residue and neutralise the charge that develops on the histidine during transition states (Polgar, 2005; Ekici, Paetzel & Dalbey, 2008). Interestingly, almost all proteins in Table 3 contained histidine as an active site to provide a proton receptor (Wang et al., 2006). In addition, Cys/His and Glu/His/Asp in peptidases also evolved convergent; however, to our knowledge, these active sites have rarely been mentioned in previous reports.

**Table 3:** Active site of convergently related peptidases.

Species	Gene	Protein	Nucleophile^a^	General base	Other active site residues
Pigs	*BLMH*	Bleomycin hydrolase	Cys73	His372	Asn396
	*AFG3L2*	AFG3-like protein 2	Glu575	His574	Asp649
	*HTRA4*	Serine protease HTRA4	Ser326	His218,	Asp248
	*CAPN7*	Calpain-7	Cys290	His458	Asn478
Humans	*OTUD5*	OTU domain-containing protein 5	Ser224	His334	Asp221
	*SENP6*	Sentrin-specific protease 6	Cys1030	His765	Asp917
	*USP14*	Ubiquitin carboxyl-terminal hydrolase 14	Cys114	His435	
	*LONP1*	Lon protease homolog, mitochondrial	Ser855	Lys898	

**Notes.**

a The number following an amino acid represents the position of the amino acid in the protein.

The analysis of housekeeping protein structure and function may reveal several interrelated and previously unrecognised relationships of structure–function constraints. These fundamental constraints have promoted the convergent evolution of housekeeping genes. Although the relationship between mechanistic convergence and functional convergence is unclear in the present study, such finding provides an entry point for our future research.

## Discussion

In the present study, we defined a set of pig housekeeping genes with a wide range of expression and low expression variation across tissues. The present set of housekeeping genes in pigs showed a lower overlap relative to the human set as the two sets showed similar physical structure and high homology. Some housekeeping genes, such as *GAPDH* and *ACTB*, in humans were not found in our list (Barber et al., 2005; De Jonge et al., 2007; Nygard et al., 2007). Thus, whether human housekeeping genes can be used as reference controls for other species remains to be verified.

After divergence from a common ancestor, pigs and humans have accumulated differences in the sequence and structure of housekeeping genes. On a molecular level, this phenomenon can occur from random mutation, for example, synonymous substitution. The dS distribution followed an approximately normal distribution, showing a random tendency for synonymous substitutions. Meanwhile, the divergence was also related to adaptive changes. In addition, GC content may affect the distribution of synonymous and non-synonymous substitutions. Hence, we also determined whether dN, dS and dN/dS of housekeeping genes were correlated with the GC content by using mouse as an outgroup. Our results showed that although a strong correlation was found between dS and GC content (*r* = 0.48, *P* = 1.94*e*^−12^), dN (*r* =  − 0.087, *P* = 0.013) and dN/dS (*r* =  − 0.11, *P* = 0.027) only showed very weak correlations with GC content. Thus, the GC content may not be the main contributing factor to the selection pressure.

Human housekeeping genes were found to be shorter than pig housekeeping genes (Figs. 3A–3C), which facilitates gene expression (Ucker & Yamamoto, 1984; Izban & Luse, 1992). In addition, the stronger purifying selection in humans than in pigs (Fig. 4A) might result in a lower degree of genetic redundancy. A source of genetic redundancy is convergent evolutionary processes, leading to genes that are close in function but unrelated in sequence, so they may also change the length of the gene structure (Zhang & Li, 2004). In other words, human housekeeping genes likely evolved more stable than pig housekeeping genes because of the advantageous and stable living environment. Moreover, humans and pigs have evolved their own species-specific housekeeping genes, which may have led to the formation of the two species, allowing the differentiated fixation of characteristics. In addition, purifying selection was stronger in common than in species-specific housekeeping genes and showed some differences in GO enrichment. This result may indicate that common housekeeping genes are more indispensable than species-specific genes and serve more functions for sustaining life. For example, *GTF2H1* (general transcription factor IIH subunit 1) and *CXXC1* (CXXC finger protein 1) in common housekeeping genes are crucial for regulating the expression of several genes (Shiekhattar et al., 1995; Butler et al., 2009), but in species-specific housekeeping genes, they were not enriched.

However, although humans and pigs have diverged for millions of years, both species independently converged towards similar features of housekeeping genes. One of the most unexpected observations was noted in species-specific housekeeping genes. GO enrichment analysis revealed that pig- and human-specific housekeeping genes serve similar functions. In addition, some housekeeping proteins evolved independently to achieve similar active sites, sidechains, catalytic centres or binding sites to complete a similar catalytic reaction or molecular function (Buller & Townsend, 2013; Polgar, 2005; Ekici, Paetzel & Dalbey, 2008; Brannigan et al., 1995; Chen et al., 2008; Klug, 2010; Klug, 1999; Hall, 2005; Brown, 2005), although these proteins showed very low homology with each other. They have ‘converged’ on the maintenance of basic cellular functions, which led to equivalent solutions for adapting to the environment (Nielsen, 2005; Hurst, 2009). Functional similarity across species may be caused by adaptive evolution (Zhang & Li, 2004; Kimura, 1983), which drives different species-specific genes to perform similar essential functions, regardless of their specific roles in the species.

At present, there is still no large-scale gene expression profile. The current transcriptome sequencing data in pigs may be inadequate to meet the requirement to define housekeeping genes. The accurate definition of housekeeping genes remains an unresolved issue. Therefore, the present set of pig housekeeping genes has limitations, but its characteristics are similar to those reported in previous studies. As new technologies emerge, high-quality deep-sequencing transcriptome profiling data may open up opportunities to improve the stringency in defining housekeeping genes and narrowing the catalogue of housekeeping genes that are expressed in a single cell (Tang et al., 2009). Furthermore, the advancement of statistical methods will greatly improve housekeeping gene detection. More specifically, the concept of ‘housekeeping’ should be defined in a hierarchical way related to cell types, growth stages, cell cycles and various physiological conditions and in terms of specific transcript variant (Zhu et al., 2008a; Zhu et al., 2008b). Thus, we will be able to observe several sets of housekeeping genes in a single species. In addition, more stringent sets of housekeeping genes will also provide powerful support for structural and functional genomics, especially for analysing the cellular basal function of different species that have some slight differences (Kumar & Hedges, 1998; Meredith et al., 2011; Kumar & Subramanian, 2002).

## Conclusions

The present study offered insight into the general aspects of housekeeping gene structure and evolution. Diverging from the ancestor of humans and pigs, housekeeping genes vary in gene structure and gene list, but they have converged to maintain basic cellular functions essential for the existence of a cell, regardless of their specific role in the species. The results in the present study will shed light on the evolutionary dynamics of housekeeping genes.

##  Supplemental Information

10.7717/peerj.4840/supp-1 Figure S1 Fraction of transcripts with expression levels being greater than a given cutoff value About 90% of the genes can be detected in our raw data.

10.7717/peerj.4840/supp-2 Figure S2 Fraction of transcripts with the *P*-value being greater than the given cutoff

10.7717/peerj.4840/supp-3 Figure S3 Distribution of dN (A) and dS (B) ratio for housekeeping genes of pigs and humans with mouse as outgroup

10.7717/peerj.4840/supp-4 Figure S4 Purifying selection on housekeeping genes with elephant as outgroup

10.7717/peerj.4840/supp-5 Figure S5 Distribution of dN (A) and dS (B) ratio for housekeeping genes of pigs and humans with elephant as outgroup

10.7717/peerj.4840/supp-6 Figure S6 Comparison of evolutionary features in human housekeeping genes with mouse as outgroup Significant differences in dN, dS and dN/dS of all, common and species-specific human housekeeping genes based on the Mann–Whitney test. In a signal cluster, all such means that share a common English letter are similar; otherwise, they differ significantly at *P* < 0.05.

10.7717/peerj.4840/supp-7 Figure S7 Comparison of evolutionary features of housekeeping genes with elephant as outgroup (A) and (B) dN, dS and dN/dS of all, common and species-specific pig and human housekeeping genes were compared based on the Mann-Whitney test. In a signal cluster, all such means that share a common English letter are similar; otherwise, they differ significantly at *P* < 0.05.

10.7717/peerj.4840/supp-8 Table S1 The information of raw data from NCBI

10.7717/peerj.4840/supp-9 Table S2 Evolutionary features of non-housekeeping genes

10.7717/peerj.4840/supp-10 Table S3 GO analysis of non-housekeeping genes

10.7717/peerj.4840/supp-11 File S1 List of pig housekeeping genes The file listed pig housekeeping genes identified in the present study.

10.7717/peerj.4840/supp-12 File S2 The dN, dS and dN/dS value of pig housekeeping genes using mouse as outgroup

10.7717/peerj.4840/supp-13 File S3 The dN, dS and dN/dS values of human housekeeping genes using mouse as outgroup

10.7717/peerj.4840/supp-14 File S4 The dN, dS and dN/dS values of pig housekeeping genes using elephant as outgroup

10.7717/peerj.4840/supp-15 File S5 The dN, dS and dN/dS values of human housekeeping genes using elephant as outgroup

## Additional Information and Declarations

### Data Availability

The following information was supplied regarding data availability: The raw data are provided in the Supplemental Tables.
