# Supplementary figures and images for: Divergent and convergent evolution of housekeeping genes in human–pig lineage
Source: PeerJ. 2018 May 24;6:e4840. doi: 10.7717/peerj.4840 (PMC5971102; doi:10.7717/peerj.4840)

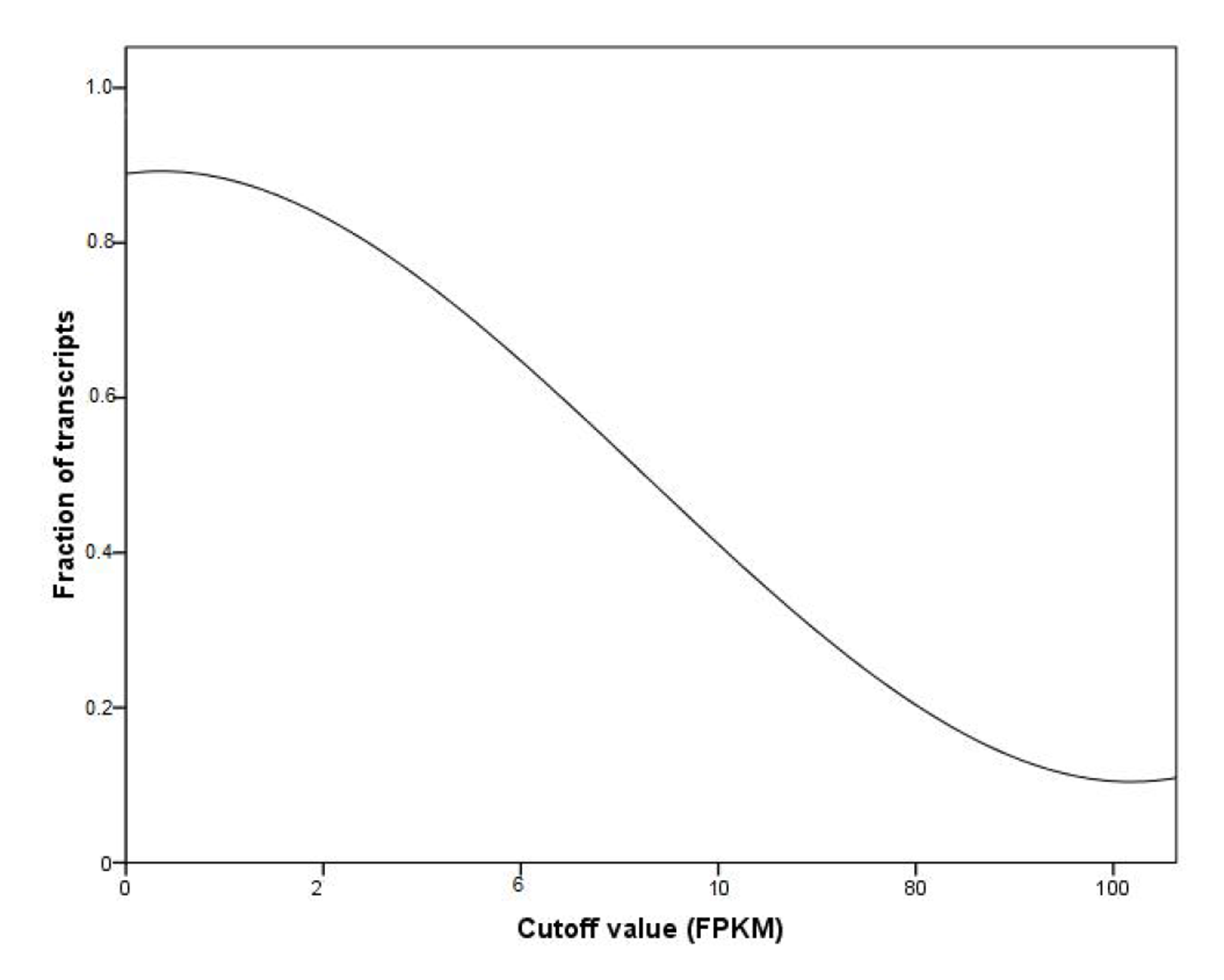

Supplement: Figure S1 — About 90% of the genes can be detected in our raw data. [file peerj-06-4840-s001.png]

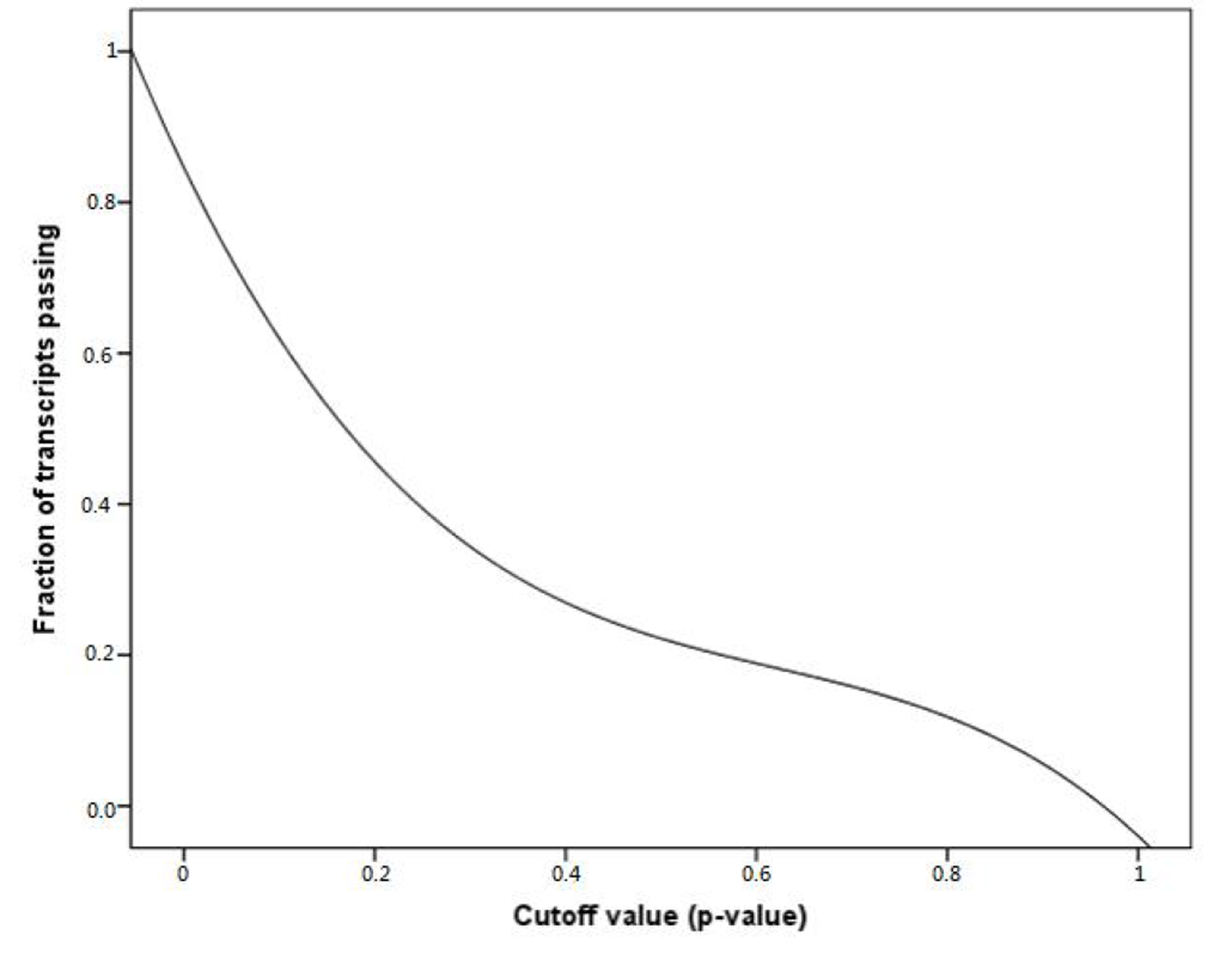

Supplement: Figure S2 [file peerj-06-4840-s002.png]

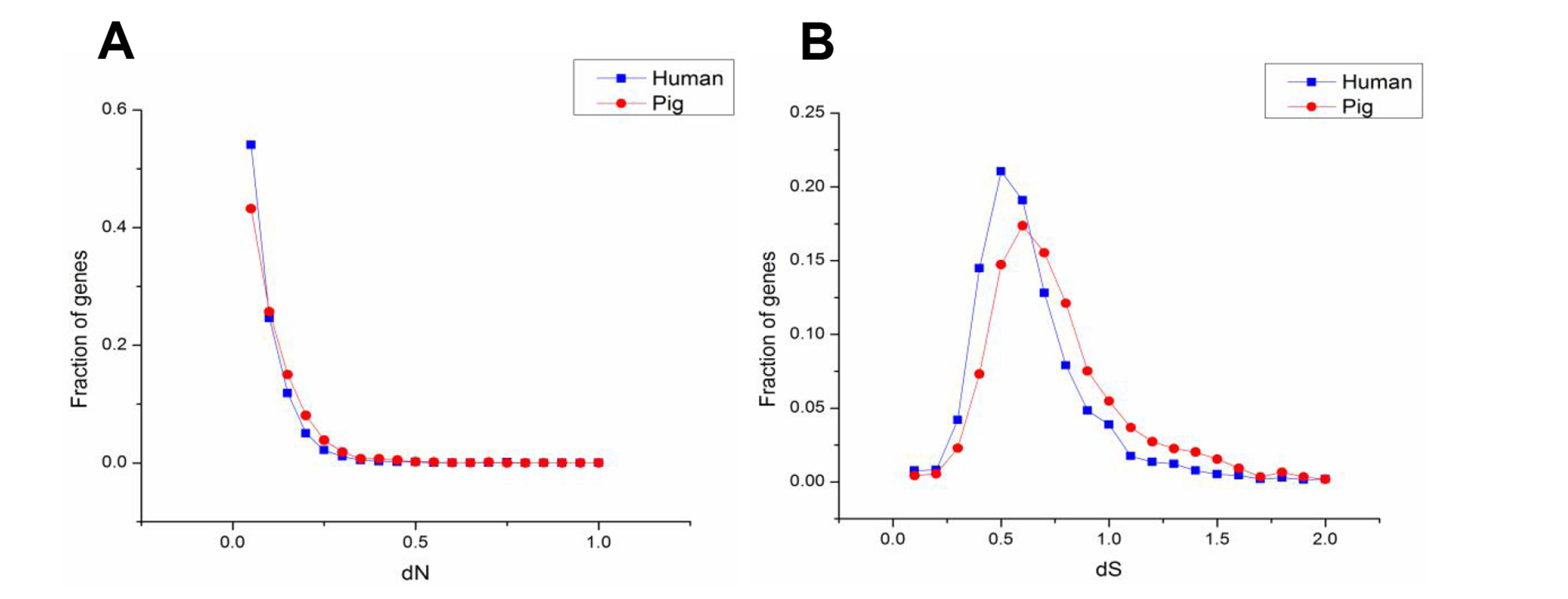

Supplement: Figure S3 [file peerj-06-4840-s003.png]

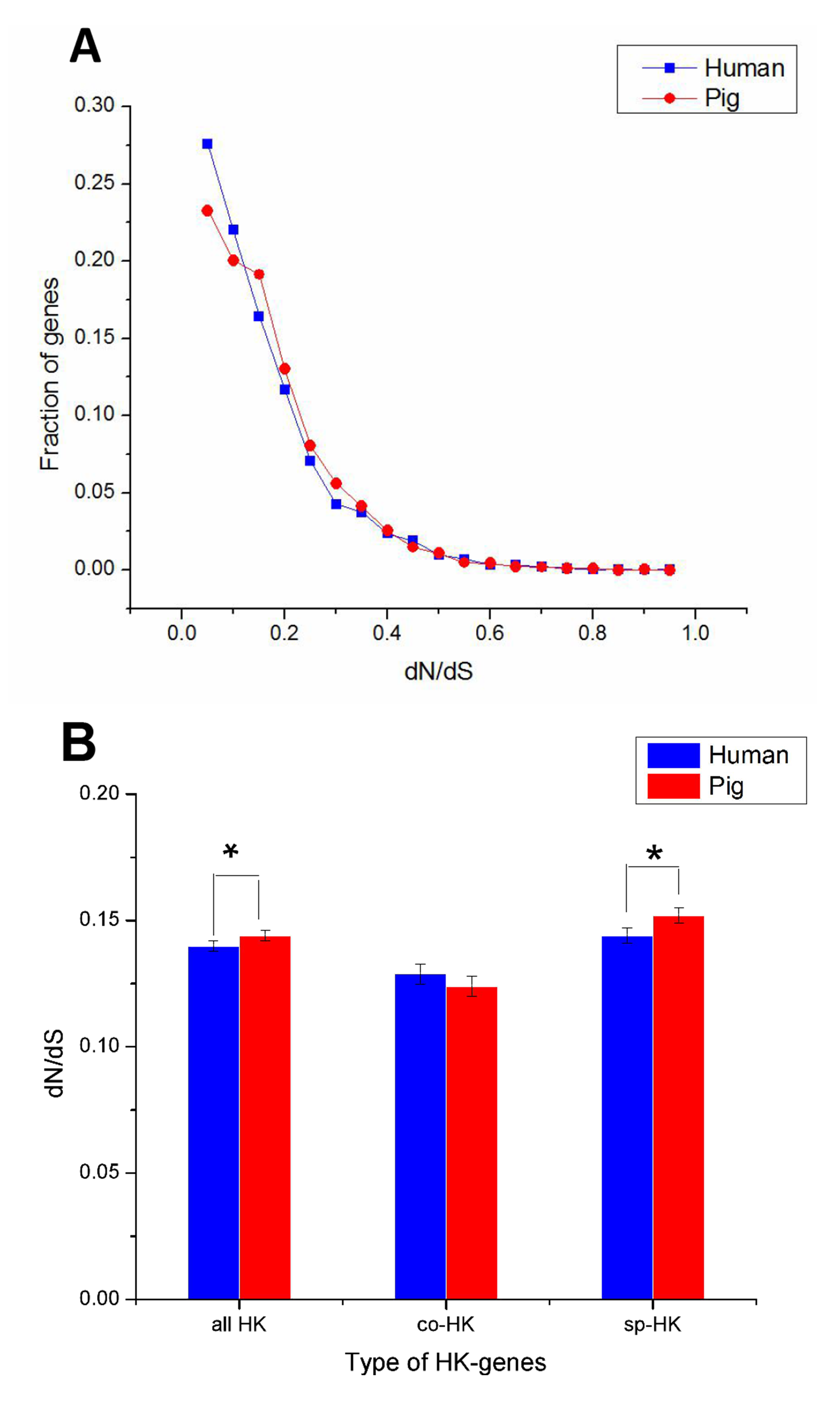

Supplement: Figure S4 [file peerj-06-4840-s004.png]

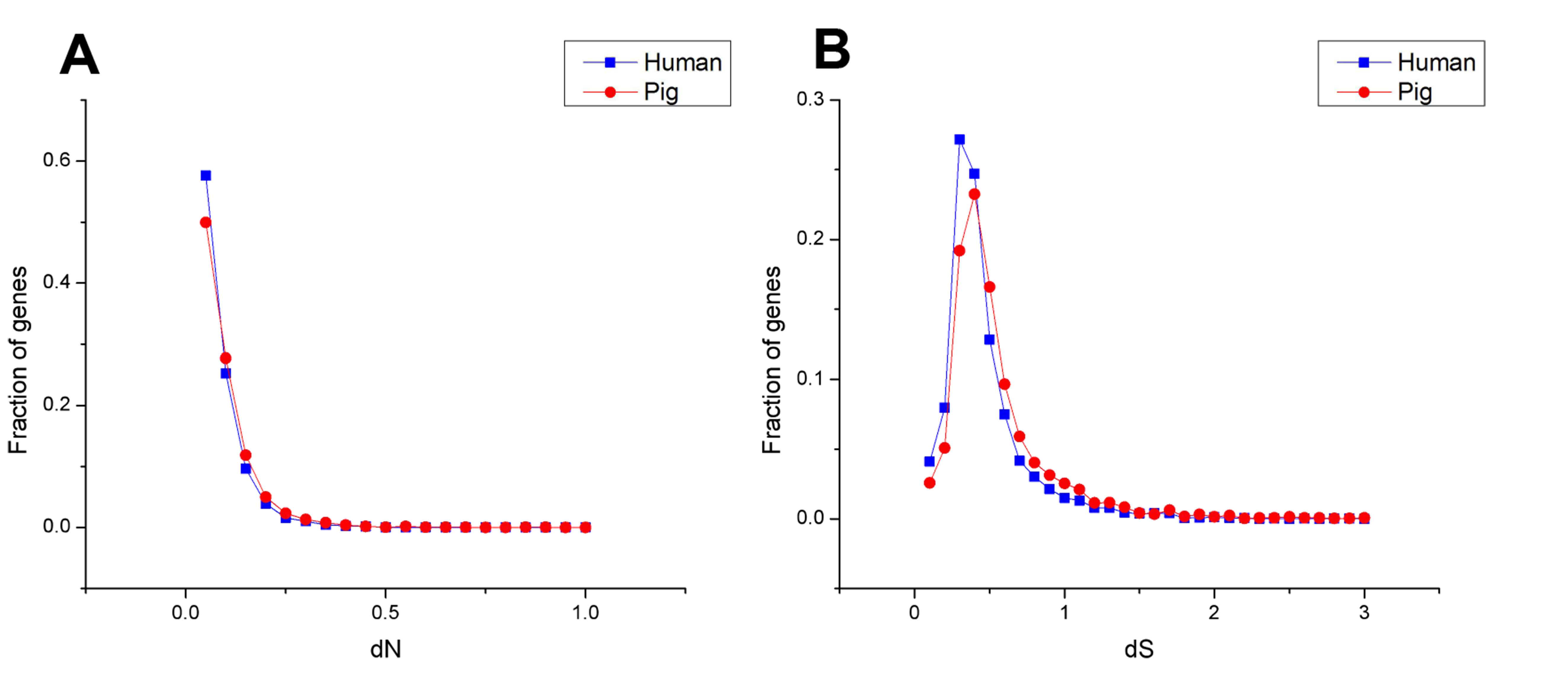

Supplement: Figure S5 [file peerj-06-4840-s005.png]

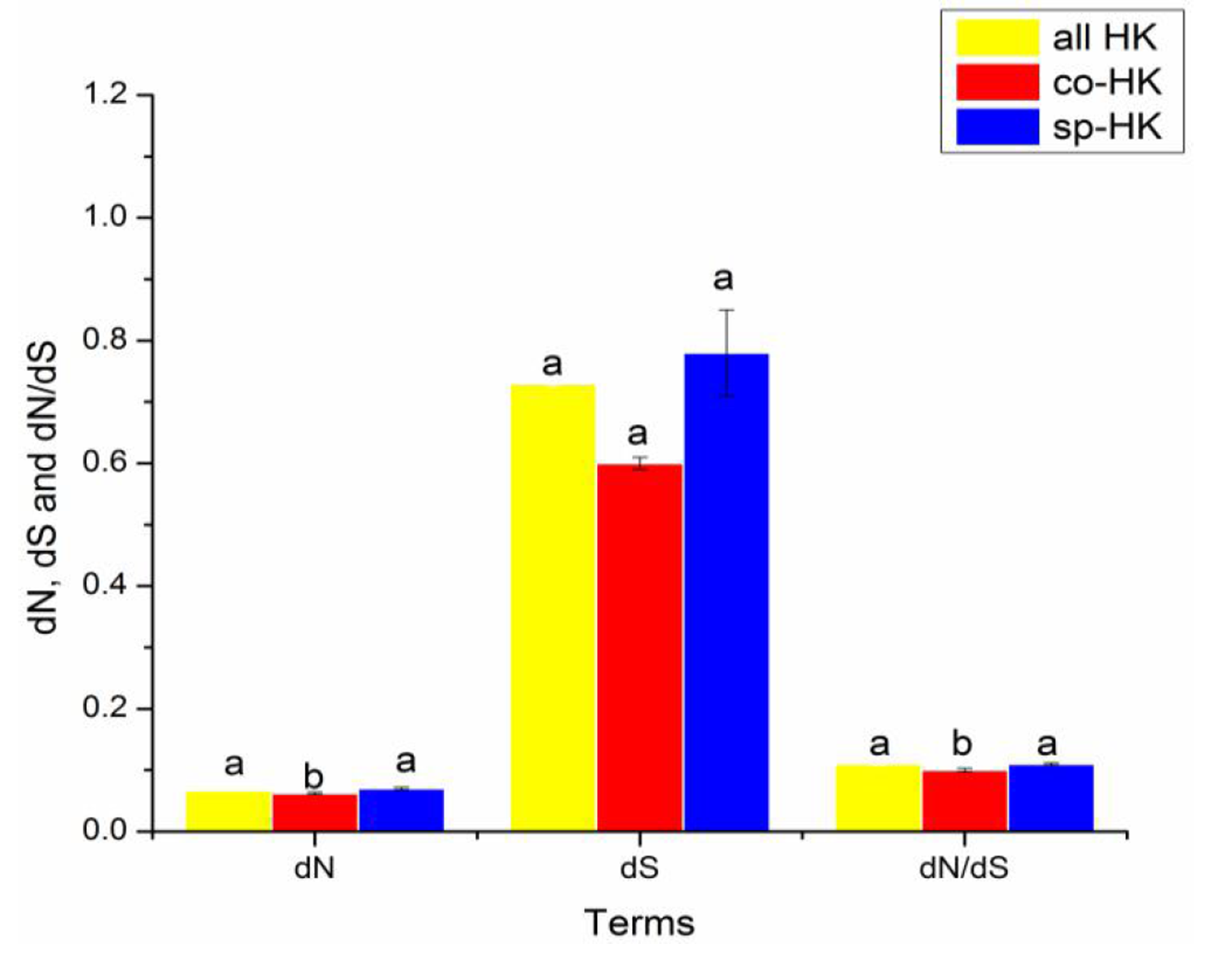

Supplement: Figure S6 — Significant differences in dN, dS and dN/dS of all, common and species-specific human housekeeping genes based on the Mann–Whitney test. In a signal cluster, all such means that share a common English letter are similar; otherwise, they differ significantly at P < 0.05. [file peerj-06-4840-s006.png]

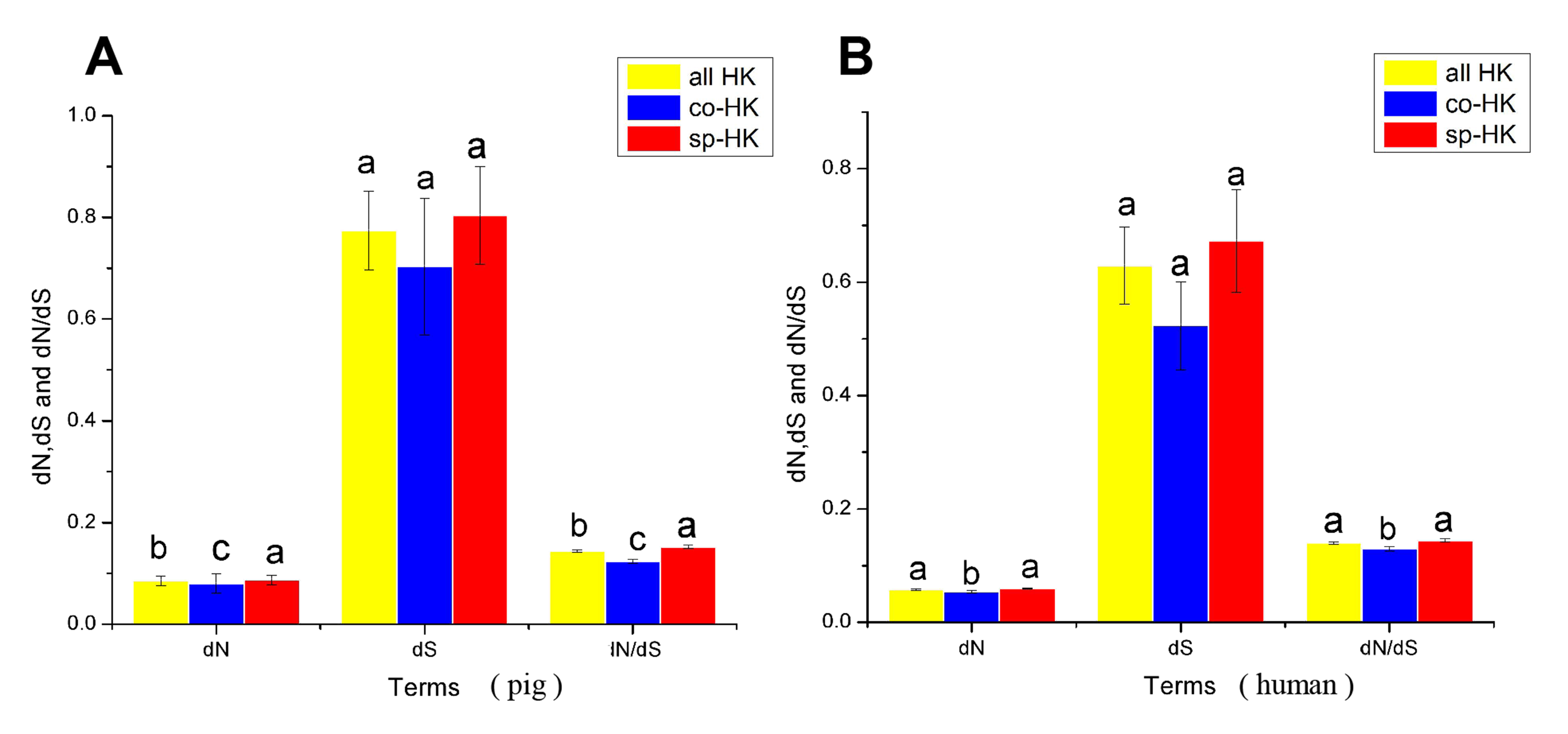

Supplement: Figure S7 — (A) and (B) dN, dS and dN/dS of all, common and species-specific pig and human housekeeping genes were compared based on the Mann-Whitney test. In a signal cluster, all such means that share a common English letter are similar; otherwise, they differ significantly at P < 0.05. [file peerj-06-4840-s007.png]
