# Supplementary material for: Divergent and convergent evolution of housekeeping genes in human–pig lineage
Source: PeerJ. 2018 May 24;6:e4840. doi: 10.7717/peerj.4840 (PMC5971102; doi:10.7717/peerj.4840)
Supplement: Table S1 [file peerj-06-4840-s008.docx]

**Table S1 The information of raw data.**

| SRA accession | Number of samples | Number of tissues | Number of reads(million) | Name of tissues | Reference |
| --- | --- | --- | --- | --- | --- |
| SRP067181 | 10 | 1 | 877 | liver | Martinez-Montes et al. 2016 |
| SRP018856 | 3 | 3 | 263 | Backfat, greater omentum, mesenterium | Wang et al. 2013 |
| SRP008516 | 2 | 1 | 50 | gonad | Esteve-Codina et al. 2011 |
| SRP032451 | 10 | 1 | 839 | hypothalamus | Pérez-Montarelo et al. 2014 |
| SRP026594 | 6 | 2 | 34 | Backfat, musculus longissimus dorsi | Jiang et al. 2013 |
| SRP018288 | 4 | 4 | 190 | heart, liver, lung, kidney | Li et al. 2013 |
| SRP018102 | 8 | 1 | 247 | endometrium | Samborski et al. 2013 |
| SRP058401 | 6 | 1 | 366 | ovary | Zhang et al. 2014 |
| SRP014902 | 3 | 3 | 59 | placenta, testis, Pool of 11 tissues | Endale Ahanda et al. 2012 |
| ERP013761 | 31 | 1 | 632 | blood | Liu et al. 2016 |
| SRP062860 | 12 | 1 | 797 | uterine | Rahman et al. 2016 |
| SRP065485 | 8 | 1 | 91 | lymph nodes | Miller et al. 2016 |
| SRP027378 | 8 | 1 | 272 | endometrium | Samborski et al. 2013 |
| ERP002055 | 20 | 10 | 223 | heart, spleen, liver, kidney, lung, musculus longissimus dorsi, occipital cortex, hypothalamus, frontal cortex, cerebellum | Farajzadeh et al. 2013 |

Endale Ahanda ML, Fritz ER, Estelle J, Hu ZL, Madsen O, Groenen MA, Beraldi D, Kapetanovic R, Hume DA, Rowland RR, Lunney JK, Rogel-Gaillard C, Reecy JM, Giuffra E. 2012. Prediction of altered 3'-UTR miRNA-binding sites from RNAseq data: the swine leukocyte antigen complex (SLA) as a model region. PLOS ONE 7(11):e48607 DOI 10.1371/journal.pone.0048607.

Esteve-Codina A, Kofler R, Palmieri N, Bussotti G, Notredame C, Perez-Enciso M. 2011. Exploring the gonad transcriptome of two extreme male pigs with RNA-seq. BMC Genomics 12:552 DOI 10.1186/1471-2164-12-552.

Jiang S, Wei H, Song T, Yang Y, Peng J, Jiang S. 2013. Transcriptome comparison between porcine subcutaneous and intramuscular stromal vascular cells during adipogenic differentiation. PLOS ONE 8(10):e77094 DOI 10.1371/journal.pone.0077094.

Li M, Tian S, Jin L, Zhou G, Li Y, Zhang Y, Wang T, Yeung CKL, Chen L, Ma J. Zhang J, Jiang A, Li J, Zhou C, Zhang J, Liu Y, Sun X, Zhao H, Niu Z, Lou P, Xian L, Shen X, Liu S, Zhang S, Zhang M, Zhu L, Shuai S, Bai L, Tang G, Liu H, Jiang Y, Mai M, Xiao J, Zhou Q, Wang Z, Stothard P, Xue M, Gao X, Luo Z, Gu Y, Zhu H, Hu X, Zhao Y, Plastow GS, Wang J, Jiang Z, Li K, Li N, Li X, Li R. 2013. Genomic analyses identify distinct patterns of selection in domesticated pigs and Tibetan wild boars. Nature Genetics 45(12):1431-1438 DOI 10.1038/ng.2811.

Liu H, Nguyen YT, Nettleton D, Dekkers JC, Tuggle CK. 2016. Post-weaning blood transcriptomic differences between Yorkshire pigs divergently selected for residual feed intake. BMC Genomics 17:73 DOI 10.1186/s12864-016-2395-x.

Martinez-Montes AM, Fernández A, Pérez-Montarelo D, Alves E, Benitez RM, Nuñez Y, Óvilo C, Ibañez Escriche N, Folch JM, Fernández AI. 2016. Using RNA-seq SNP data to reveal potential causal mutations related to pig production traits and RNA editing. Animal Genetics 48(2):151-165 DOI 10.1111/age.12507.

Miller LC, Bayles DO, Zanella EL, Lager KM. 2016. Effects of pseudorabies virus infection on the tracheobronchial lymph node transcriptome. Bioinformatics and Biology Insights 9(Suppl 2):25-36 DOI 10.4137/BBI.S30522.

Pérez-Montarelo D, Madsen O, Alves E, Rodriguez MC, Folch JM, Noguera JL, Groenen MA, Fernández AI. 2014. Identification of genes regulating growth and fatness traits in pig through hypothalamic transcriptome analysis. Physiological Genomics 46(6):195-206 DOI 10.1152/physiolgenomics.00151.2013.

Rahman KM, Camp ME, Prasad N, McNeel AK, Levy SE, Bartol FF, Bagnell CA. 2016. Age and nursing affect the neonatal porcine uterine transcriptome. Biology of Reproduction 94(2):46 DOI 10.1095/biolreprod.115.136150.

Samborski A, Graf A, Krebs S, Kessler B, Bauersachs S. 2013a. Deep sequencing of the porcine endometrial transcriptome on day 14 of pregnancy. Biology of Reproduction 88(4):84 DOI 10.1095/biolreprod.113.107870.

Samborski A, Graf A, Krebs S, Kessler B, Reichenbach M, Reichenbach HD, Ulbrich SE, Bauersachs S. 2013b. Transcriptome changes in the porcine endometrium during the preattachment phase. Biology of Reproduction 89(6):134 DOI 10.1095/biolreprod.113.112177.

Trapnell C, Williams BA, Pertea G, Mortazavi A, Kwan G, Van Baren J, Salzberg SL, Wold BJ, Pachter L. 2010. Transcript assembly and quantification by RNA-seq reveals unannotated transcripts and isoform switching during cell differentiation. Nature Biotechnology 28(5):511-515 DOI 10.1038/nbt.1621.

Wang T, Jiang A, Guo Y, Tan Y, Tang G, Mai M, Liu H, Xiao J, Li M, Li X. 2013. Deep sequencing of the transcriptome reveals inflammatory features of porcine visceral adipose tissue. International Journal of Biological Sciences 9(6):550-556 DOI 10.7150/ijbs.6257.

Zhang X, Huang L, Wu T, Feng Y, Ding Y, Ye P, Yin Z. 2015. Transcriptomic analysis of ovaries from pigs with high and low litter size. PLOS ONE 10(1):e0139514 DOI 10.1371/journal.pone.0139514.
