# Supplementary material for: Divergent and convergent evolution of housekeeping genes in human–pig lineage
Source: PeerJ. 2018 May 24;6:e4840. doi: 10.7717/peerj.4840 (PMC5971102; doi:10.7717/peerj.4840)
Supplement: Table S2 [file peerj-06-4840-s009.docx]

**Table S2 Evolutionary features of non-housekeeping genes**

| Terms | Mouse*^a^* | | |  | Elephant | | |
| --- | --- | --- | --- | --- | --- | --- | --- |
|  | Pig | Human | *P*-value *^c^* |  | Pig | Human | *P*-value |
| dN | 0.16±0.419 *^b^* | 0.15±0.027 | 0.410 |  | 0.12±0.012 | 0.13±0.019 | 0.295 |
| dS | 1.39±6.093 | 1.91±8.061 | 0.013 |  | 1.54±7.459 | 0.84±3.926 | 0.001 |
| dN/dS | 0.17±0.018 | 0.20±0.032 | 0.894 |  | 0.21±0.27 | 0.24±0.42 | 0.175 |
