# Supplementary material for: Divergent and convergent evolution of housekeeping genes in human–pig lineage
Source: PeerJ. 2018 May 24;6:e4840. doi: 10.7717/peerj.4840 (PMC5971102; doi:10.7717/peerj.4840)
Supplement: Table S3 [file peerj-06-4840-s010.docx]

| GO terms | Pig (%) | Human (%) |
| --- | --- | --- |
| positive regulation of epithelial cell proliferation | 1.43 | 1.36 |
| G-protein coupled receptor signaling pathway | 7.16 | 7.93 |
| cardiovascular system development | 5.11 | 5.75 |
| osteoblast differentiation | - | 1.36 |
| nervous system development | 13.62 | 12.79 |
| cellular response to hormone stimulus | 2.68 | 3.97 |
| protein complex assembly | 10.06 | 8.74 |
| regulation of osteoblast differentiation | 0.79 | 1.01 |
| regulation of actin cytoskeleton organization | 2.14 | 1.87 |
| regulation of blood coagulation | 1.17 | 0.82 |
| skin development | 1.37 | 1.24 |
| brain development | 3.98 | 4.16 |
| synaptic signaling | - | 3.69 |
| transmembrane transport | 6.53 | 8.04 |
| lung development | 0.89 | 1.13 |
| secretion | 5.46 | 6.34 |
| glial cell differentiation | - | 1.32 |
| lymphocyte activation | 3.74 | 3.34 |
| negative regulation of protein metabolic process | 6.74 | 5.67 |
| immune system development | 4.82 | 4.59 |
| skeletal system morphogenesis | - | 1.32 |
| eye development | 1.36 | 1.98 |
| reproductive system development | 3.47 | 2.49 |
| muscle cell differentiation | 1.34 | 1.75 |
| muscle organ development | - | 2.09 |
| embryonic organ development | 2.17 | 2.18 |
| vasculature development | 3.68 | 4.04 |

Table S3 GO analysis of non-housekeeping genes
